# Reply to Pavlik et al. Clinical Relevance and Environmental Prevalence of *Mycobacterium fortuitum* Group Members. Comment on “Mugetti et al. Gene Sequencing and Phylogenetic Analysis: Powerful Tools for an Improved Diagnosis of Fish Mycobacteriosis Caused by *Mycobacterium fortuitum* Group Members. *Microorganisms* 2021, *9*, 797”

**DOI:** 10.3390/microorganisms10010055

**Published:** 2021-12-28

**Authors:** Davide Mugetti, Mattia Tomasoni, Paolo Pastorino, Giuseppe Esposito, Vasco Menconi, Alessandro Dondo, Marino Prearo

**Affiliations:** Istituto Zooprofilattico Sperimentale del Piemonte, Liguria e Valle d’Aosta, Via Bologna 148, 10154 Torino, Italy; mattia.tomasoni@izsto.it (M.T.); paolo.pastorino@izsto.it (P.P.); giuseppe.esposito@izsto.it (G.E.); vasco.menconi@izsto.it (V.M.); alessandro.dondo@izsto.it (A.D.); marino.prearo@izsto.it (M.P.)

We appreciate the valuable comment of Pavlik et al. [1] for our previous manuscript. This comment enhances the attention on an emerging problem, such as infections and distribution of the less known non-tuberculous mycobacteria. With regard to that, the authors focused on literature regarding the members of the *Mycobacterium fortuitum* group (MFG), including works both concerning environmental distribution and cases of clinical interest. The interest in these microorganisms has certainly increased in relation to various factors.

Firstly, the use, to a greater extent, of novel diagnostic techniques (e.g., gene sequencing, MALDI-TOF mass spectrometry) has made it possible to have tools to reach an ever-greater level of discrimination at species and subspecies level for several bacterial genera. As in the case of non-tuberculous mycobacteria, other microorganisms are also difficult to identify at the species level using biochemical tests or sequencing of the gene encoding the 16S subunit of ribosomal RNA (universal method used for bacterial taxonomy). Regarding 16S rRNA for species determination, limits have been reported for several genera including *Aeromonas*, *Nocardia*, and *Vibrio*, in addition to the already mentioned fast growing mycobacteria [2,3,4,5]. Reasonably, the major limits that are linked to this technique are found for environmental or recently classified microorganisms, for which a limited number of sequences are available in databases. Therefore, the use of different housekeeping genes with greater variability compared to 16S rRNA (e.g., *hsp65*, *rpoB*) or techniques that cover a greater genome portion (e.g., WGS) allow an increasingly higher level of identification for environmental/lesser-known species, assuming that sequences are available [6,7,8].

Secondly, there is a constant increase in the number of cases of diseases caused by bacteria that were historically known as environmental microorganisms. The genus *Mycobacterium*, as reported by Pavlik et al. [1] regarding MFG and for other species in previous works [9], presents several examples to support this statement; however, there are many other cases that are referable to other bacterial genera. An example is *Stenotrophomonas maltophilia*: previously known as *Pseudomonas maltophilia* [10], later as *Xanthomonas maltophilia* and only in 1993 classified as we know it today, it is a bacterium that is found in the natural environment (water, sediment, soil, plants) [11]. The increase in cases of immunocompromised patients, the high antibiotic resistance of different *S. maltophilia* strains, and the use of techniques that allow univocal identification have made it possible to identify this bacterium more frequently in clinical practice [12,13]. Other similar cases are represented by *Burkholderia cepacia* complex, *P. aeruginosa*, and *Ochrobactrum* spp., all opportunistic pathogens that can be isolated from environmental samples [14,15,16]. Therefore, it is important to diagnose these bacteria with effective techniques both in cases of infection in human/animals and in their natural environments [17].

In conclusion, it is conceivable that there will always be a greater number of reports of environmental and less-known microorganisms from a clinical point of view in the future. In the case of retrospective studies, emerging pathogens are likely to be found in the case of application of molecular diagnostic techniques, as is the case of our MFG study [18]. Studies of this type are recommended to understand if the “new pathogens” are present in previous clinical samples or if they are a really emerging problem. Furthermore, it will be necessary to focus on the diagnosis by applying a One Health perspective, considering any animal and environmental reservoirs, in addition to the human patient.

Finally, we want to thank again Pavlik and co-authors for having constructively commented on our work, providing a further perspective on the sources of isolation of MFG microorganisms.
